# Physiotherapy Intervention for a 1.5-Year-Old Child With Communicating Hydrocephalus and Developmental Delay Secondary to Torticollis: A Case Report

**DOI:** 10.7759/cureus.70260

**Published:** 2024-09-26

**Authors:** Shivani R Dethe, H. V. Sharath, Pratiksha A Warghat, Raghumahanti Raghuveer

**Affiliations:** 1 Department of Pediatric Physiotherapy, Ravi Nair Physiotherapy College, Datta Meghe Institute of Higher Education and Research, Wardha, IND; 2 Department of Neuro-Physiotherapy, Ravi Nair Physiotherapy College, Datta Meghe Institute of Higher Education and Research, Wardha, IND

**Keywords:** communicating hydrocephalus, developmental delay, pediatrics, physical therapy, torticollis

## Abstract

This report presents the case of a 1.5-year-old female child diagnosed with communicating hydrocephalus and developmental delay, who received physical therapy as part of her treatment. Hydrocephalus refers to the formation of excess fluid in the deep brain cavities. The excess fluid makes the ventricles enlarged, which increases the pressure inside the brain. Excessive cerebrospinal fluid (CSF) pressure caused by hydrocephalus, on the other hand, can harm brain tissue and cause a variety of neurological problems and developmental delays. After taking a thorough history in this case, it was discovered that the patient had a history of one-month NICU admissions due to frequent episodes of vomiting, fever, and low birth weight. After repeated delays, medical consultations were requested, and a magnetic resonance imaging (MRI) showing signs of communicating hypertension led to a referral for physiotherapy. The child shows delays in reaching developmental milestones, such as having difficulty controlling her head and trunk and having a history of consequent birth complications. The goals of the physiotherapy intervention were to develop total body coordination and balance, improve awareness of sensations, and attain head and trunk control. Different approaches were used to target developmental milestones and functional abilities, such as myofascial release techniques, neurodevelopmental approaches, sensory stimulation, and integration therapy. The patient's outcome measures were evaluated both before and after the intervention using the Hammersmith infant neurological examination, Face, Legs, Activity, Cry, and Consolability scale, and infant neurological international battery. All outcome measures indicated significant improvements after receiving physiotherapy rehabilitation. Significant improvements were achieved through progressive treatments, demonstrating the importance of combining early therapy and parental engagement in assisting patients in achieving their goals. To investigate additional therapy methods and evidence of long-term prognosis for this patient population, more study is required.

## Introduction

Hydrocephalus is a condition characterized by an excess of cerebrospinal fluid (CSF) created inside the central nervous system (CNS) as a result of an irregularity in CSF production and absorption [1]. While the exact etiology of some types of hydrocephalus may remain unknown, other types might be associated with factors such as hemorrhage, infection, or trauma that block CSF flow and induce ventricular expansion [2]. Higher intracranial pressure (ICP) is often associated with acute hydrocephalus, which can cause symptoms such as headaches, vomiting, nausea, abnormalities of the cranial nerves, changes in consciousness, and, in the worst case, death or coma [3]. There are still a few exceptions to the generalization about chronic hydrocephalic diseases. These rare cases include idiopathic normal pressure hydrocephalus (NPH), which usually affects individuals and is associated with normal ICP levels, and unknown chronic hydrocephalus [4].

The clear, colorless fluid that surrounds and supports the brain and spinal cord is called cerebrospinal fluid. After the CSF covers the brain and spinal cord and passes through the ventricles, it is reabsorbed into the bloodstream. The choroid plexus, which is part of the brain's lateral ventricles, produces CSF through ependymal cells. Communicating hydrocephalus is the result of a blockage of CSF after it exits the ventricles. Conditions including as meningitis, subarachnoid hemorrhage, and congenital or acquired diseases may be the cause of this blockage [5]. One kind of non-obstructive communicative hydrocephalus that can be caused by trauma or idiopathic conditions is NPH [6]. Since non-communicating hydrocephalus results from an intraventricular occlusion of CSF flow, it is invariably of the obstructive type. Among the obstruction regions are the cerebral aqueduct and the foramen of Monro [7].

According to pathophysiology, CSF dynamics change throughout the development of hydrocephalus. CSF exchange is limited by the third ventricles and the lateral ventricles. Hydrocephalus causes dilation of the foramen of Monro. As the lateral ventricle and the third ventricle separate into mono ventricles, the cerebral aqueduct continues to operate [8]. The clinical features of hydrocephalus are significantly influenced by the patient's age and the rate of improvement [9]. Before the cranial sutures close (before the child reaches two years of age), hydrocephalus manifests clinically as macrocephaly, an increase in head circumference, regression, poor nutrition, developmental delay, enlarged veins of the scalp, and sunset sign. The acute appearance after the cranial sutures close can include papilledema, headache, vomiting, and, in the most severe cases, coma. Treatment options for congenital and acquired hydrocephalus include neuroendoscopy and shunt surgery [10].

The two main surgical procedures are right ventriculoperitoneal (VP) shunting and endoscopic third ventriculostomy, which is a surgical technique that can help prevent shunt-related complications such as shunt obstruction, infection, and overdraining. To perform VP shunting, a distal catheter is put into the peritoneal cavity and a proximal catheter is introduced into the cerebral ventricle. In a developing child, torticollis may result from biomechanical malfunction of the shunt material connected to tethering at the most movable portion of the shunt tube (neck). This is the report of the VP shunt problem in the literature, as far as we are aware. The only incidence of comparable consequences included a patient who experienced torticollis as a result of a fibrous band that formed down the length of a previous VP shunt that had been removed four years prior [10,11].

Children with hydrocephalus have impairments in their posture, strength, balance, walking, and fine motor abilities [12]. The development of a child's cognitive, social, emotional, and language skills follows from an understanding of both gross and fine motor skills [13]. Preventing a greater developmental motor delay requires a physiotherapy intervention that maximizes neurodevelopmental techniques and encourages the child to attain developmental motor milestones [14].

## Case presentation

A 1.5-year-old female child with hydrocephalus with VP shunt in situ presented with complaints of vomiting since morning and fever. On August 9, 2024, the mother observed that she had an episode of non-projectile vomiting containing food particles, as well as fever associated with chills and rigor. The child also had decreased appetite since then, was irritable, and had a staring look. Hence, the child was brought to Acharya Vinoba Bhave Rural Hospital (AVBRH) for further management. The child also had a history of NICU stay for one month under observation given hydrocephalus. The child has a history of multiple admissions for surgery and similar complaints in the past, with the last admission being in February 2024. According to the primary caregiver, the child was born on December 2, 2022. At the age of one month, the child had an episode of fever and vomiting after breast feeding, for which they visited a private hospital for further management. The weight of the baby was reduced to 1.5 kg. Further investigation was conducted, which revealed the hydrocephalus. The patient was then referred to the PICU of AVBRH for one month. On January 23, 2023, the child underwent subglacial pouch insertion and then the baby was discharged. In April 2023, the child was brought with complaints of accidentally pulling out her subgleal pouch, and then on July 13, 2023, the child underwent right-sided VP shunting and removal of the subgleal pouch. At the age of 12 months, she came with complaints of episodes of vomiting and fever; hence, after further medical management on February 22, 2024, she underwent endoscopic adhesiolysis and left ventriculoperitoneal shunt (Kocher’s point) placement. MRI of the brain revealed multiple signal-intensity loculated cystic lesions noted in bilateral cerebral hemispheres (left frontal, left parietal, bilateral occipital, right temporoparietal) adjacent to the third ventricle and right cerebellar hemisphere adjacent to the fourth ventricle.

Clinical findings

Informed consent from the patient’s caregiver was obtained before the clinical examination, which was followed by the physical examination. Compared to her developmental age, the patient did not meet any developmental milestones. Table 1 below provides a comprehensive timeline of the event.

**Table 1:** Timeline of events AVBRH, Acharya Vinoba Bhave Rural Hospital

Events	Timeline
Date of birth	December 2, 2022
Was diagnosed with communicating hydrocephalus	December 2, 2022
Date of first operation	January 23, 2023
Date of second operation	July 13, 2023
Date of third operation	February 22, 2024
Visited AVBRH in response to the following concerns	August 10, 2024
An assessment for physiotherapy is done	August 23, 2024

Developmental milestones associated with gross motor was not achieved, as will be discussed below. A comprehensive summary of gross motor development is presented in Table 2.

**Table 2:** Gross motor skills associated with developmental milestones

Gross motor skills	Typical range	Attained month
Head control	6 weeks	Partial head control is achieved in one year, but after surgery, head control is lost
Rolling	4-6 months	Partial rolling
Sitting	5-7 months	Not achieved
Creeping	6-8 months	Not achieved
Crawling	9-11 months	Not achieved
Standing with support	9-12 months	Not achieved
Walking with support	10-15 months	Not achieved

Table 3 shows developmental milestones related to fine motor skills. While other skills, such as grasp and transfers, were not developed within the necessary time frame, the grasp reflex was developed at eight months, reach reflex at 12 months, release reflex at nine months, and mouthing reflex at 12 months.

**Table 3:** Developmental milestones relevant to fine motor skills

Fine motor skills	Typical range	Attained month
Grasp reflex	0-3 months	8 months
Reach	2-4 months	12 months
Release	3-6 months	9 months
Mouthing	3-6 months	12 months
Transfers	4-6 months	Not achieved
Grasp	6-8 months	Not achieved

Language-related milestones are shown in Table 4. At eight months, the baby could turn her head to sound, at six months she started cooing, and at 12 months she started uttering monosyllables. However, at nine months, she was still unable to produce disyllables.

**Table 4:** Language-related milestones

Language	Normal	Attained month
Turns head to sound	6 weeks	7 months
Cooing	3 months	8 months
Monosyllables	6 months	12 months
Diasyllables	9 months	Not achieved

Developmental milestones related to social and personal communication are shown in Table 5. While a social smile and mother recognition were attained at seven and nine months, respectively, smiling at a mirror image and waving bye-bye were not attained at six or nine months.

**Table 5:** Developmental milestones related to personal and social communication

Personal and social	Normal	Attained month
Social smile	1 month	9 months
Recognizing mother	3 months	7 months
Smiles at a mirror image	6 months	Not achieved
Waves bye bye	9 months	Not achieved
Plays a simple ball game	12 months	Not achieved

Investigation 

**Figure 1:**
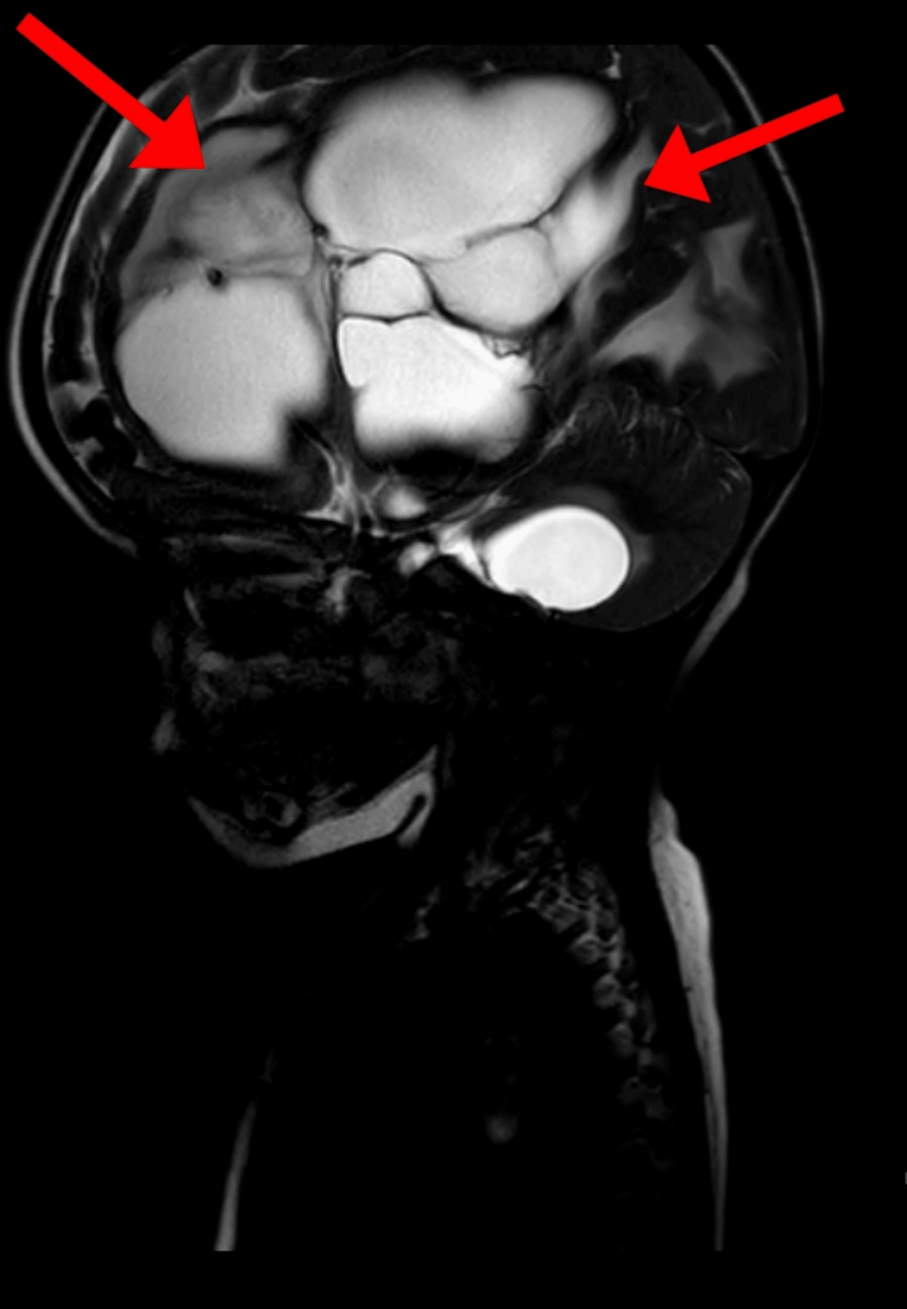
MRI of the brain showing multiple signal intensity loculated cystic lesions noted in bilateral cerebral hemispheres (left frontal, left parietal, bilateral occipital, right temporoparietal) adjacent to the third ventricle and right cerebellar hemisphere adjacent to the fourth ventricle.

The MRI of the brain demonstrated multiple cystic lesions with varying signal intensities. These loculated lesions were seen bilaterally across the cerebral hemispheres, including the left frontal, left parietal, bilateral occipital, and right temporoparietal regions. Additionally, a lesion was located near the third ventricle, as well as a cystic lesion in the right cerebellar hemisphere adjacent to the fourth ventricle, as illustrated in Figure 1. The appearance of these cystic lesions may indicate a pathological process such as infection, congenital malformations, or other cystic brain abnormalities that require further clinical correlation.

Physiotherapy intervention

A comprehensive rehabilitation program with daily hour-long sessions was put into place, as shown in Table 6. In addition, the primary caregiver provided suggestions for activities that might be done at home.

**Table 6:** Physiotherapy intervention

Goals	Intervention	Dosage
To reduce neck tightness and shortness	Stretching of the sternocleidomastoid muscle of the left side. Myofascial release techniques include kneading and stroking in circular motion bilaterally to release muscular tightness and muscular shortness.	10 repetitions x 1 set with 10 seconds hold, 3 repetitions x 1 set for 30 seconds
To improve neck control	Stabilizing the head and neck allows for bouncing on the Swiss ball. To gently stroke the back of the neck, lie prone on a peanut ball, roll forward, and wait for the child to lift their head against gravity. Progressing in a pull-to-sit position while stretching the intercostal muscles. On a Swiss ball, prone on hands and elbows. Shifting the weight while seated on a tilt board.	6 days per week, 1-2 minutes of exercise with 2 minutes of rest for a total of 10 minutes of session.
To improve trunk mobility	Rotation of the trunk when lying supine. Trunk rotation and stretching when seated and lying down. After stabilizing the child's pelvis, the upper body rotated to hold positions to the right and left at the end range. Rotate a trunk in both directions while maintaining pelvic stability in the supine position and vice versa.	30 seconds to 1 minute, three sets
To facilitate rolling	Rolling on a dynamic surface like a Swiss ball. Stabilizing the upper and lower limbs. The exercise was initially performed on a static mat or a flat surface.	1-2 minutes, followed by 2 minutes of break. For 5-8 minutes, each side was trained.
To improve trunk control	Swiss ball activities include bouncing over the ball with little support above the waist and moving the ball in all directions, such as moving the infant backward on the ball and waiting for the child's reaction to come forward using the trunk/core muscles. When a child is in a standing position, apply minimal perturbations.	6 days per week, 1-2 minutes of activity with 2 minutes of rest. Each side turning trained for 5-8 minutes.
To facilitate quadruped position	To increase upper limb strength along with extension of the elbow. Prone on the elbow, then advance to prone on the hand and weight-bearing on the upper limb with a bolster and wedge.	2-3 minutes with 2 minutes of rest, with a total of 15-20 minutes
To facilitate tracking of objects	Because the child preferred bright lights, he or she was placed in a dark room, with a flashing light projected onto the wall or ceiling to improve visual tracking. To create various colors of light, use different colored plastic filters.	5-10 minutes

Outcome measures

Using a variety of outcome measures, the study assessed gross motor functions, neurological examination findings, and the condition of the musculoskeletal system both before and after physiotherapeutic procedures. The pre- and post-physiotherapy intervention results are shown in Table 7, which shows a significant improvement in each of the assessed parameters.

**Table 7:** The pre and post-physiotherapy intervention outcomes FLACC, Face, Legs, Activity, Cry, and Consolability

Outcome measures	Pre-intervention	Post-intervention
Hammersmith infant neurological examination	30	45
FLACC scale	5/10	8/10

## Discussion

The excessive amount of CSF in the brain can result in hydrocephalus. Birth problems can cause hydrocephalus, which can either be inherited or developed later in life. Communicating, non-communicating, and ex vacuo are types of hydrocephalus in different forms. A diagnosis is typically made via a physical examination and diagnostic imaging. Around one or two neonates out of 1,000 are affected by hydrocephalus. The developed world may have greater rates. Five people out of 100,000 are affected by NPH, and the frequency increases with age [15].

Since hydrocephalus patients are at a greater risk of delays in both physical and mental growth, continual rehabilitation management is necessary for cases involving pediatric patients [16]. A physiotherapy rehabilitation plan for a 1.5-year-old female patient with communicating hydrocephalus is described in this case. When compared to normal developmental timelines, the patient's developmental milestones were noticeably delayed. When infants exhibit clinically delayed developmental milestones, it frequently raises questions about possible neurological malfunction [17]. In this case, brain MRI reveals multiple signal-intensity loculated cystic lesions in bilateral cerebral hemispheres (left frontal, left parietal, bilateral occipital, right temporoparietal) adjacent to the third ventricle and right cerebellar hemisphere adjacent to the fourth ventricle.

The first developmental milestone attained by individuals is head control. If not attained, it may cause the remaining milestones to decline [18]. To develop both static and dynamic activity, the baby was trained on a bolster, a Swiss ball, and support at the trunk. As the activity advanced to quadruped, the infant's head control was facilitated without the need for trunk support. The same techniques are used to enhance balance and trunk muscular control [19]. Studies indicate that alternate approaches to Bobath or neurodevelopmental therapy in neurorehabilitation are effective in enhancing motor function despite their conventional application in these circumstances [20]. Physiotherapy treatment in this case study included the tightness in the neck, mobilization of soft tissues, myofascial release methods (kneading, stroking), and neurodevelopmental techniques. To meet the patient's requirements, neurodevelopmental approaches, sensory integration therapy, and sensory stimulation were applied. The duration of each session was modified based on the patient's phase of development, and this intervention was given six days a week.

All outcome measures showed significant improvements throughout physiotherapy rehabilitation. These outcomes highlight how effectively physical therapy rehabilitation can treat developmental delays brought about by communicating hydrocephalus [21]. To evaluate developmental outcomes, address new concerns, and adjust treatment plans according to requirements, long-term follow-up is necessary [22,23].

## Conclusions

This case report emphasizes the significance of physical therapy intervention for communicating hydrocephalus in a child who is 1.5 years old and has poor head and trunk control. A improvement in motor function was made possible by prompt diagnosis and suitable treatment, such as physiotherapy. Physiotherapy rehabilitation is mostly responsible for improving the activity of living and optimizing results for affected children. Further investigation and clinical trials are necessary to examine long-term effects and other treatment alternatives for the current patient population.
